# Characterizing the “sweet spot” for the preservation of a T-cell line using osmolytes

**DOI:** 10.1038/s41598-018-34638-7

**Published:** 2018-11-01

**Authors:** Chia-Hsing Pi, Guanglin Yu, Ashley Petersen, Allison Hubel

**Affiliations:** 1 Department of Mechanical Engineering, University of Minnesota, Minneapolis, 55455 USA; 2 Division of Biostatistics, University of Minnesota, Minneapolis, 55455 USA

## Abstract

This study examined the post-thaw recovery of Jurkat cells cryopreserved in single osmolyte solutions containing sucrose, glycerol or isoleucine, as well as in a combination of the three osmolytes. Cell response was determined using low temperature Raman Spectroscopy and variation in post-thaw recovery with composition was analyzed using statistical modeling. Post-thaw recovery of Jurkat cells in single osmolyte was low. A combination of the osmolytes displayed a non-linear relationship between composition and post-thaw recovery, suggesting that interactions exist between the different solutes. The post-thaw recovery for an optimized multicomponent solution was comparable to that observed using 10% dimethyl sulfoxide and a cooling rate of 1 °C/min. Statistical modeling was used to characterize the importance of each osmolyte in the combination and test for interactions between osmolytes. Higher concentrations of glycerol increase post-thaw recovery and interactions between sucrose and glycerol, as well as sucrose and isoleucine improve post-thaw recovery. Raman images clearly demonstrated that damaging intracellular ice formation was observed more often in the presence of single osmolytes as well as non-optimized multi-component solution compositions.

## Introduction

Over the past several years, immunotherapy has emerged and been called the “fourth pillar” of cancer treatment. Chimeric antigen receptor (CAR) T-cell therapy is a rapidly growing therapy for the treatment of cancer^1^. The U.S. Food and Drug Administration (FDA) approved two CAR T-cell therapies in 2017, Kymriah developed by Novartis for the treatment of children with acute lymphoblastic leukemia and Yescarta developed by Kite for adults with advanced lymphomas. Further progress with the use of immunotherapies for the treatment of cancer as well as other diseases is also anticipated.

Dimethyl sulfoxide (DMSO) has been the standard cryopreservation agent for freezing cells since the 1960 s^2^. However, DMSO is toxic upon infusion to patients and can lead to side effects from mild (such as nausea and vomiting) to severe (such as cardiovascular) or even cause death^3^. When exposed to DMSO, cells lose viability and function with time of exposure^4^. For hematopoietic cells, exposure to DMSO is typically limited to 30 min^5^. This practice adds to the complexity of the workflow associated with preservation of cells using DMSO.

There is a demand for DMSO-free cryoprotectants that maintain cell viability and function after thaw. Diverse biological systems (plants, insects, etc.) survive high salt environments, dehydration, drought, freezing temperatures and other stresses through the use of osmolytes^6^. In the human kidney, a mixture of five osmolytes are used to stabilize the cells^7^. Recently we developed a method of preserving cells with combinations of osmolytes^8–10^. These studies demonstrated that a combination of three different osmolytes including sugar, sugar alcohol and amino acids/proteins could stabilize Jurkat cells and mesenchymal stromal cells (MSCs) during freezing. Each of the components plays a role in stabilization of the cell during freezing. Sugars are associated with stabilization of the cell membrane^11^ and interaction via hydrogen bonding with water^12^, thereby changing solidification patterns. Glycerol also interacts strongly with water^13^ via hydrogen bonds, penetrates the cell membrane^14^ and is associated with stabilization of proteins^15^. Amino acids help stabilize sugars during freezing so that they do not precipitate out of solution^16^. It is noteworthy that higher levels of osmolytes did not necessarily correspond to higher post-thaw viability^17^. The osmolytes appeared to act in concert to improve post-thaw recovery.

The objective of this investigation is to understand in more detail the relationships amongst the osmolytes present in these solutions and Jurkat cell recovery. Raman spectroscopy has been widely used in characterizing subcellular structures such as mitochondrion, lysosome and nucleus because it is label-free and has high spatial resolution^18^. Moreover, Raman spectroscopy can identify the phase of water (liquid or solid) and the location of cryoprotective agents. For this study, low temperature Raman spectroscopy was used to interrogate freezing responses of cells cryopreserved in different combinations of osmolytes. This tool enables us to quantify intracellular ice formation (IIF), distribution of cryoprotective agents, damage to subcellular compartments and other cell behaviors during freezing^17,19^.

In a previous study, we demonstrated that osmolytes act in concert to improve cell viability^17^. A recent study demonstrated that combinations of osmolytes had a strong effect on crystallization of water and form natural deep eutectic systems (NADES)^20^. The next phase of the investigation will involve characterizing the role of a given osmolyte and its interactions with other osmolytes on post-thaw recovery using a statistical model. This type of analysis will provide the foundation for a molecular model of protection and osmolyte interaction. This knowledge is critical for the development of improved cryopreservation protocols, in particular, for high value cells such as cell therapies.

## Materials and Methods

### Cell culture

Jurkat cells (ATCC TIB-152), a T-cell line, whose identity was confirmed by Short Tandem Repeat (STR) profiling were used in this investigation. Jurkat cells are a model cell line for T-cells and have also been used the production of IL-2 and studies of T-cell receptor signaling^18^. The cells were cultured in high-glucose RPMI 1640 (Life Technologies, Carlsbad, CA, USA) with 10% fetal bovine serum (FBS; Qualified, Life Technologies, Carlsbad, CA, USA). Cultures were maintained at densities ranging between 1 × 10^5^ and 3 × 10^6^ cells/mL. Cells for Raman spectroscopy were prepared by washing and centrifuging cells twice in Dulbecco’s Phosphate Buffered Saline at 125 × g for 10 min. Cells were then resuspended in the experimental solution of interest and frozen using a thermally controlled stage described below.

### Toxicity studies

Cryopreservation solutions are typically not physiological and exposure to the solutions can result in cell losses. In order to determine the toxicity of the candidate solutions, Jurkat cells were exposed to candidate solutions at room temperature. Viability of the cells was determined at different time points post exposure. The highest acceptable cell losses were set to 10% (90% viability). Cells were incubated in 96-well plates (Corning, NY, USA) for all candidate solutions. Test solutions were made at 2× of their final concentration in Normosol-R (Hospira). Cells were centrifuged and resuspended in Normosol-R and then combined 1:1 with the 2× solution, using a single-step addition in clear-bottom black 96-well plates to produce a 1× concentration of cryoprotectant solution with a total volume of 50 μL and a cell concentration of 300,000 cells/well (6 million cells/mL). Calcein acetoxymethyl (Calcein-AM, Life Technologies) and propidium iodine (PI, Life Technologies) were used to determine viability. Calcein-AM/PI dye was added to each well at a 1:1 ratio between dye and tested solution volume. After addition of the dye, the plates were wrapped in aluminum foil to protect from light exposure and incubated for a half hour at 37 °C and CO_2_ at 5 vol%. The fluorescence of each plate was read at 530/590 nm and 485/528 nm. A control curve was obtained by reading plates with known numbers of live and dead cells in each well. The fluorescence readings for an experimental plate were compared to the control curve for correlating the amount of live and dead cells in each well. All experimental studies were performed in sextuplicate wells on each plate.

### Freezing experiments

Cells were frozen in 96-well plates (Corning, NY, USA) for all studies using the same procedure as the toxicity studies. Cells were frozen in 10% DMSO as a control. All experimental studies were performed in triplicate wells on each plate. The cells were incubated in the solutions of interest for one hour at room temperature in the plates before being sealed with silicone round well covers (Laboratory Supply Distributers, Millville, NJ, USA) to prevent desiccation during freezing and storage.

All samples were cryopreserved using a controlled-rate freezer (Series III Kryo 10; Planer, Middlesex, UK). The plates were placed in a plastic rack in a controlled-rate freezer, and frozen using the following profile: (1) start at 20 °C, (2) −10 °C/min to 0 °C, (3) hold at 0 °C for 15 min, (4) −1 °C/min to −8 °C, (5) −50 °C/min to −45 °C, (6) +15 °C/min to −12 °C, (7) −1 °C/min to −60 °C, and (8) −10 °C/min to −100 °C. The rapid cooling and rewarming (steps 5 and 6) helped to nucleate ice in the extracellular solution. After the freezing procedure was completed, plates were stored in the vapor phase of liquid nitrogen until thawed.

### Thawing and post-thaw assessment

Thawing was performed in a 37 °C water bath, and thawing was complete in less than 3 min. The post-thaw staining of live/dead cells with Calcein-AM/PI was as same as in the toxicity studies. The post-thaw recovery was defined as the ratio of the number of live cells post-thaw to the number of seeded live cells.

### Osmolarity

Osmolarity of solutions were measured using an OSMETTE^TM^ osmometer (Precision Systems, Natick, MA) for each solution and all measurements were repeated in triplicate.

### Raman spectroscopy and thermally controlled stage

Confocal Raman spectroscopy measurements were conducted using a Confocal Raman Microscope System Alpha 300R (WITec, Ulm, Germany) with a UHTS300 spectrometer and DV401 CCD detector with 600/mm grating. The WITec spectrometer was calibrated with a Mercury-Argon lamp. A Nd:YAG laser (532 nm wavelength) was used as an excitation source. A 100× air objective (NA 0.90; Nikon Instrument, Melville, NY) was used for focusing the 532 nm excitation laser to the sample. The laser at the objective was 10 mW, as measured by an optical power meter (Thorlabs, Newton, NJ). The lateral resolution of the microscope was about 296 nm according to Abbe’s diffraction formula. Cell samples were frozen using a four-stage Peltier (Thermonamic Electronics Corp. Jiangxi, China) and a series 800 temperature controller (Alpha Omega Instruments Corp, Lincoln, RI). Cell samples were seeded at −6 °C with a liquid nitrogen cooled needle, cryopreserved at 1 °C/min to a holding temperature of −50 °C and held for 20 min before imaging. Condensation was minimized by creating a barrier around the imaging stage using plastic film (Bemis, Neenah, WI) and purging the space with dry nitrogen gas. About 1–3 µL of cell suspension was placed on the stage, covered with a piece of mica (TED PELLA, Redding, CA) and sealed with Kapton tape (Dupont, Wilmington, DE) to prevent sample evaporation/sublimation.

### Raman image/spectra analysis

**Figure 1:**
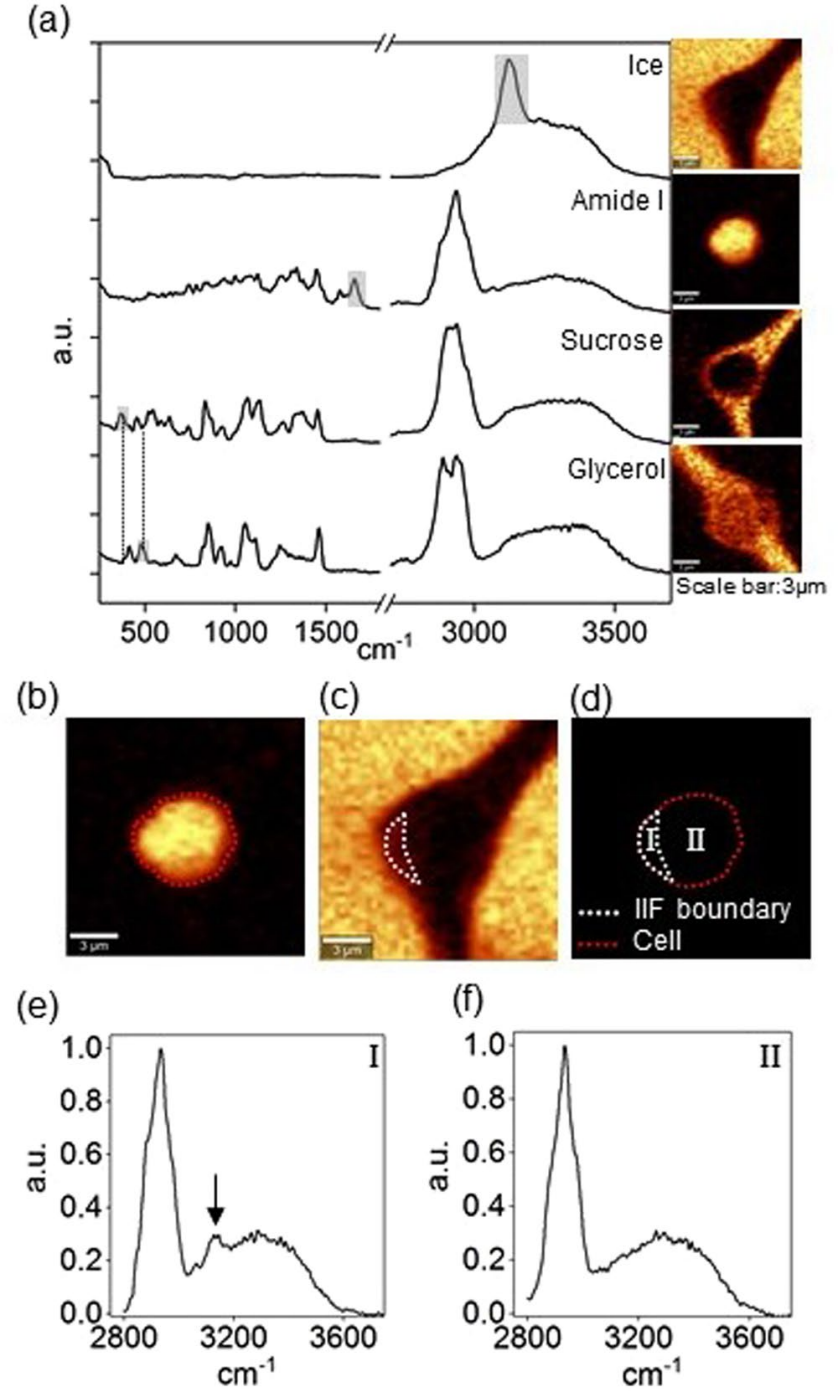
(**a**) Raman spectra and images of ice, amide I, sucrose, and glycerol. Raman images were rendered based on the specific Raman signals indicated on the spectra. The gray area indicates the peak used to generate corresponding Raman images. (**b**) Raman image of amide I showing cell boundary. (**c**) Raman image of ice showing IIF boundary. (**d**) IIF and cell boundary for AIC calculation. Region I and II represented the cellular portion with or without IIF, respectively. (**e**) Raman spectra of cell section with IIF. The arrow indicates Raman signal of ice crystal. (**f**) Raman spectra of cell section without IIF.

Raman images were generated by integrating spectrums at each pixel based on characteristic wavelength of common intracellular and extracellular materials (Fig. 1a). Raman signals and the associated wavenumbers selected for these studies are given in Table 1. Amide I and Alkyl C=C stretches were used to generate distribution of protein and lipid to delineate the area of frozen Jurkat cells. Images of ice were generated with background subtraction at both sides of the peak range to separate ice and water signals. The image size was 15 µm × 15 µm and each image had 45 × 45 pixels with an integration time of 0.2 sec for each pixel. The Raman signals used this study did not overlap with each other; as a result, multivariate data analysis was not required. Cell boundary was determined by applying contour function on Raman image of amide I in WITec Project FOUR software (Fig. 1b). IIF was determined by the presence of OH stretch peak at 3125 cm^−1^. Raman spectra of cell section with IIF showed presence of OH stretch peak, while Raman spectra of cell section without IIF showed absence of OH stretch peak (Fig. 1c–f). The ratio of cross-sectional area of IIF to the cross-sectional area of cell was calculated in ImageJ and termed as area of ice-to-cell (AIC) in the following text.

**Table 1:** Wavenumber Assignments for Raman Spectra.

Substance	Wavenumber cm^−1^	Assignments^19,44,48^
Ice	3125	OH stretching
Protein and Lipid (Cell)	1660	Amide I and Alkyl C = C stretching
Sucrose	375	COC bending
Glycerol	476	CCO bending

### Statistical analysis

Mean plus/minus standard error was reported for all measurements unless otherwise noted. Two-tailed Student’s *t*-tests were performed for two-sample comparisons to obtain *p*-values. Statistical modeling was performed using R, version 3.4.0 (https://www.R-project.org/) for Windows OS.

The variation in post-thaw recovery with composition was modeled using a quasi-binomial model. Two models were fit: (1) a main effects model to quantify the influence of each osmolyte and (2) a model with interactions to test for pairwise interactions between osmolytes. The main effects model included predictors for the concentration levels of sucrose, glycerol and isoleucine. The interaction model included the main effects plus the three pairwise interactions between sucrose, glycerol and isoleucine. In both models, the concentration level of sucrose was modeled as a categorical variable in order to allow the possibility of a non-monotonic relationship, as was observed in the single component study.

## Results

### Single component studies

Initially, the variation of cell survival as a function of solution composition was determined for single component (sugar, sugar alcohol and amino acid). The concentration of a given osmolyte was varied from 0% to 100% of the solubility limit or alternatively the toxicity limit for the cell to screen the space with all possible formulations.

Preliminary toxicity studies were performed to determine the parameter space for the single component study. Cell losses >10% were considered unacceptable and the upper-level of cryoprotective agents were based on that level of acceptable cell losses. For concentrations of sucrose above 730 mM, cell losses with time increased rapidly after 1-hour incubation, but cell loss was still acceptable for 2190 mM for 1-hour incubation and the upper threshold of single component studies for sucrose was set at 2190 mM (Supplementary Fig. S1a). Cell losses in glycerol were high for all concentrations above 10% and for times greater than one hour (Fig. S1b) and as a result, the upper threshold of glycerol concentration was set at 10%. The viability of Jurkat cells in isoleucine was independent of concentration and incubation time (Supplementary Fig. S1c) and the upper limit of isoleucine used was based on the solubility limit. It is noteworthy that Jurkat cells incubated in SGI155 exhibited minimal losses over the 4-hour period studied (Supplementary Fig. S1d).

**Figure 2:**
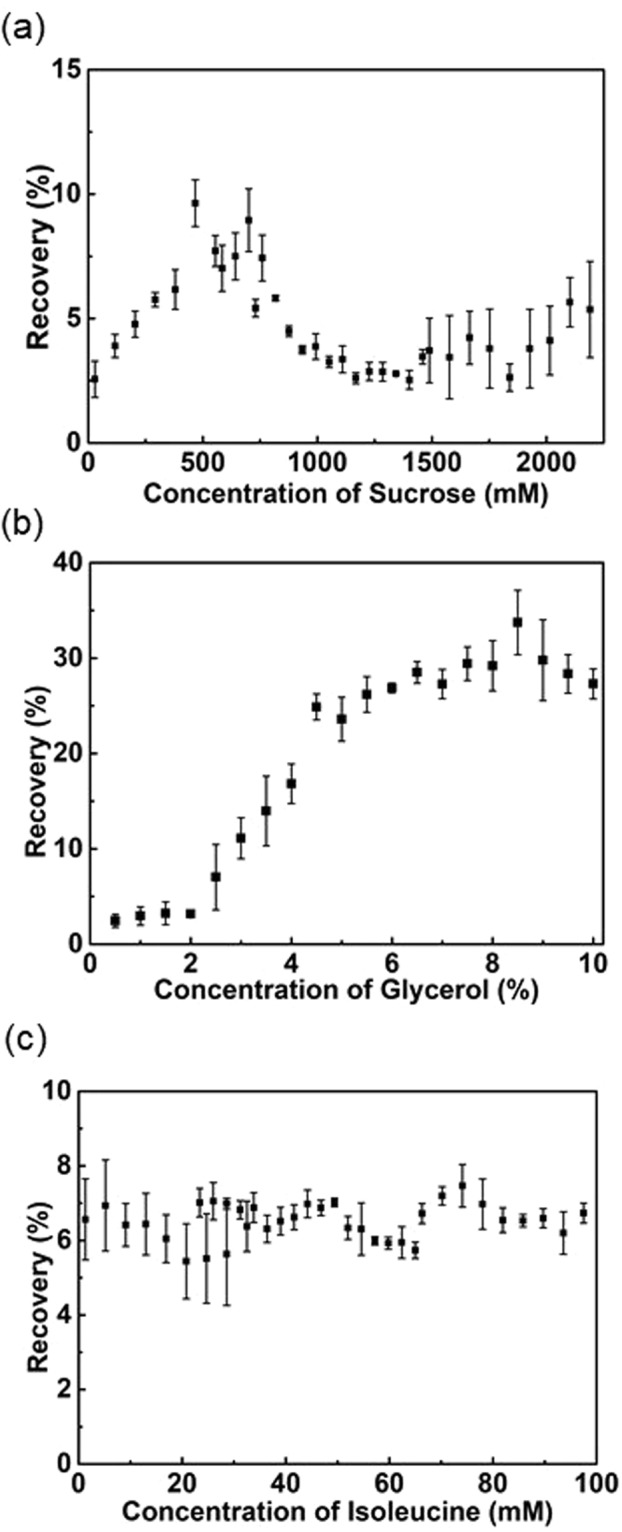
Post-thaw recoveries of Jurkat cells cryopreserved at −1 °C/min as a function of (**a**) sucrose concentration; (**b**) glycerol concentration; and (**c**) isoleucine concentration.

Post-thaw recovery for Jurkat cells in sucrose varied between roughly 3% and 10% over the range of concentrations based on toxicity studies (Fig. 2a). The cooling rate for single component studies was 1 °C/min according to previously published work^9^. The maximum post-thaw recovery occurred at roughly 730 mM. In contrast, the post-thaw recovery of cells cryopreserved in glycerol increased with increasing concentration to a threshold concentration of ~8% (Fig. 2b) and achieved a maximum recovery of 40%. The post-thaw recovery of cells cryopreserved in isoleucine was low (~7%) and remained largely unchanged across the range of tested concentrations (Fig. 2c).

**Figure 3:**
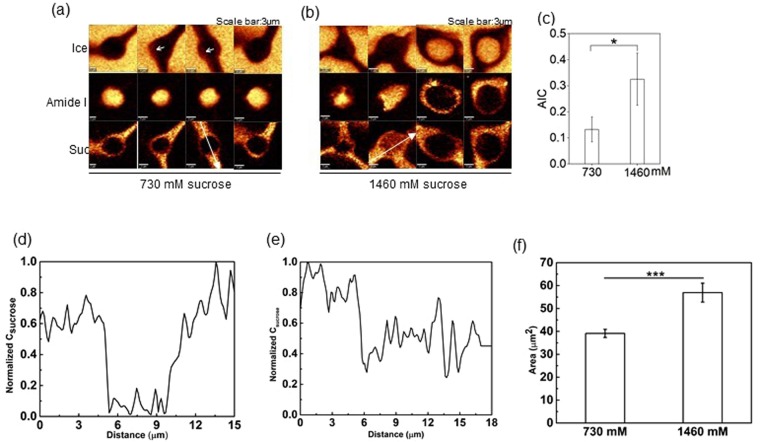
(**a**) Raman images of ice, amide I, and sucrose of cells cryopreserved in 730 mM sucrose solution. (**b**) Raman images of ice, amide I, and sucrose of cells cryopreserved in 1460 mM sucrose solution. (**c**) AIC of cells cryopreserved in 730 mM and 1460 mM sucrose solution (n = 8, p = 0.033). (**d**) Normalized concentration of sucrose along the white arrow in (**a**). (**e**) Normalized concentration of sucrose along the white arrow in (**b**). (**f**) Cross-sectional area of cells cryopreserved in 730 mM and 1460 mM (n = 8, p < 0.001). (**g**) Raman images of ice, amide I, and glycerol of cells cryopreserved in 4% glycerol solution.

As indicated in Fig. 2a, the recovery of Jurkat cells cryopreserved in sucrose solutions varied with concentration. To explore the effects of sucrose concentration on the freezing response of cells, Jurkat cells in 730 mM and 1460 mM sucrose solution were cryopreserved at a constant cooling rate of 1 °C/min down to −50 °C, and Raman images rendered on the signals associated with ice, amide I and sucrose were generated (Fig. 3a,b). Cells cryopreserved in 730 mM sucrose solution showed small ice crystals (indicated by the white arrow in the image of ice) based on the presence of OH stretching peak. In contrast, large pieces of pure ice crystals were observed in the center of cells cryopreserved in 1460 mM sucrose solution (3 out of 8 cells). Accordingly, AIC of cells cryopreserved in 1460 mM sucrose solution was significantly greater than that of cells cryopreserved in 730 mM sucrose solution (Fig. 3c). For cells cryopreserved in 730 mM sucrose solution, Raman images showed that sucrose was predominantly distributed in the unfrozen solution, forming a thin layer encircling the frozen cell (<1 μm) (Fig. 3d). For cells cryopreserved in 1460 mM sucrose solution, substantial penetration of sucrose into cells was detected in five of the eight cells studied, suggesting cell membrane of those cells was possibly damaged (Fig. 3e). Raman images of amide I also showed that cells cryopreserved in 730 mM sucrose solution maintained normal but smaller cell size. The cross-sectional area of 1460 mM sucrose (57 µm^2^) was significantly larger (p = 0.009) than 730 mM sucrose (39 µm^2^) (Fig. 3f) once again suggesting damage to the cell membrane. On the contrary, cells cryopreserved in 1460 mM sucrose solution showed irregular cell shape.

### Multicomponent studies

#### Variation in response with cooling rate

Cooling rate is a key factor in post-thaw recovery and the composition can also influence the optimum cooling rate. As a result, the influence of cooling rate on post-thaw recovery to multicomponent solutions was determined before screening the entire operation space. Eight formulations spanning the extremes of the parameter space (level 0 or level 5 of a given component) and 10% DMSO were tested with three cooling rates (1 °C/min, 3 °C/min and 10 °C/min). The post-thaw recoveries were higher at 1 °C/min than those observed at 3 °C/min and 10 °C/min for the formulations tested (Supplementary Fig. S2). As a result, a cooling rate of 1 °C/min was used for subsequent experiments.

#### Post-thaw recovery of multicomponent solutions

The concentration limit of sucrose was truncated to 730 mM (the peak of post-thaw recovery) based on the single component freezing studies, glycerol was limited to 10% and isoleucine was limited to 43 mM based on the toxicity studies described above. The concentration space of each component was discretized to six levels with equal scale (216 formulations total, Table 2). The actual composition was described using these levels. For example, 353 was the combination of level-three sucrose, level-five glycerol and level-three isoleucine. The post-thaw recovery as a function of composition was determined across all 216 formulations.

**Table 2:** Definition of concentration level and corresponding absolute concentration for the components tested.

	Sucrose (mM)	Glycerol (%)	Isoleucine (mM)
Level 0	0	0	0
Level 1	146	2	8.67
Level 2	292	4	17.33
Level 3	438	6	26.00
Level 4	584	8	34.67
Level 5	730	10	43.33

Post-thaw recovery was plotted as a function of osmolarity for different combinations of sucrose, glycerol and isoleucine tested (see Supplementary Fig. S3). Over a range of osmolarity from 200 to 1600 mOsm/kg, there was little correlation between post-thaw recovery and osmolarity (R^2^ = 0.2293). This result is consistent with what we have observed previously with other cell types^17^.

**Figure 4:**
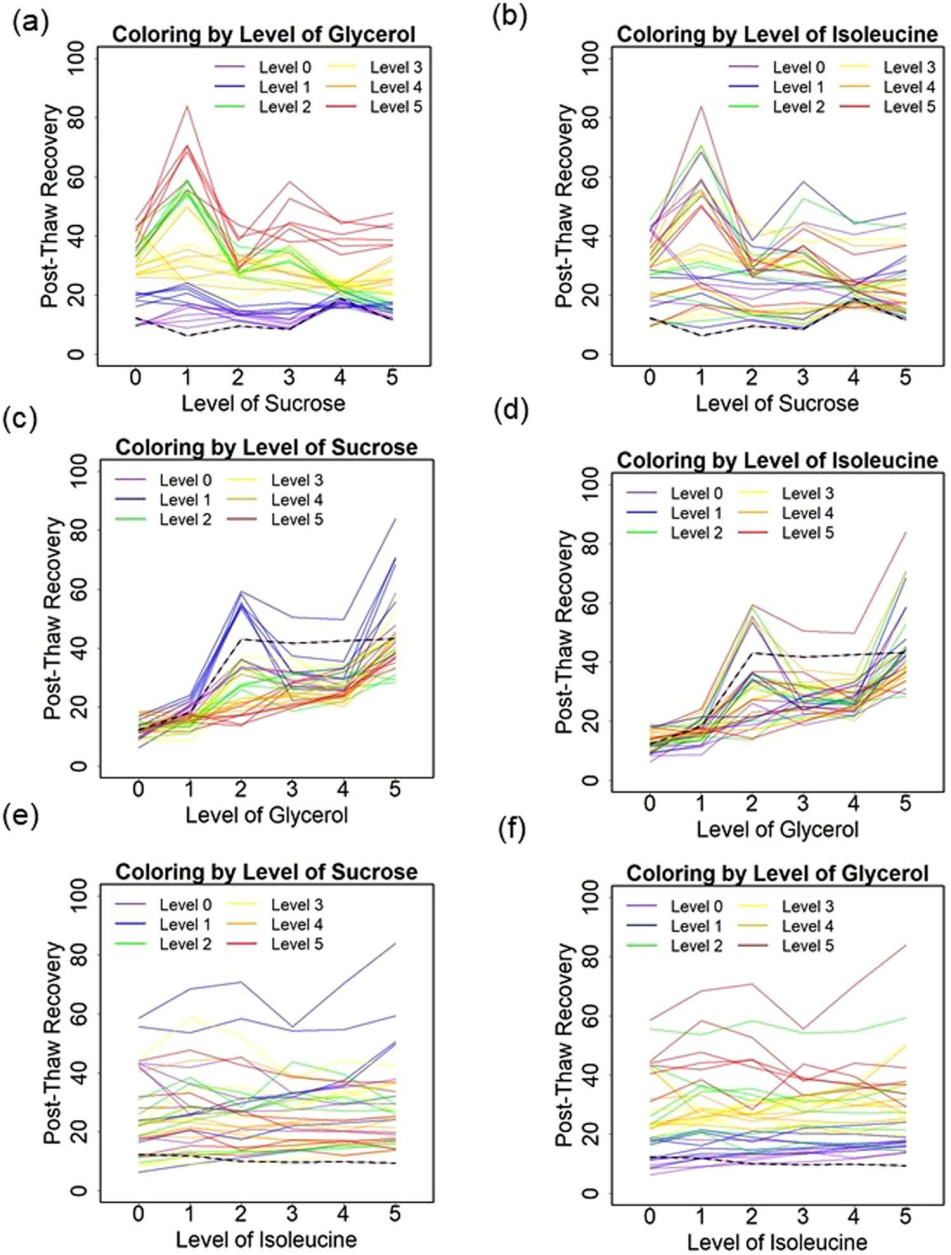
Post-thaw recoveries of Jurkat cells cryopreserved at a cooling rate of 1 °C/min and plotted to show (**a**) the effect of sucrose with coloring by level of glycerol, (**b**) the effect of sucrose with coloring by level of isoleucine, (**c**) the effect of glycerol with coloring by level of sucrose, (**d**) the effect of glycerol with coloring by level of isoleucine, (**e**) the effect of isoleucine with coloring by level of sucrose, and (**f**) the effect of isoleucine with coloring by level of glycerol. Each solid line demonstrates the effect of the x-axis osmolyte on post-thaw recovery for fixed levels of the other two osmolytes. The dashed lines indicate the post-thaw recoveries for the single component solutions.

The optimal formulation was the combination of 146 mM sucrose (level 1), 10% glycerol (level 5) and 46 mM isoleucine (level 5) solution (SGI155) with 84% post-thaw recovery. To visualize the interactions between osmolytes, spaghetti plots of sucrose, glycerol, isoleucine and post-thaw recovery were presented (Fig. 4). Each subfigure showed a plot of the mean post-thaw recovery vs concentration level of one osmolyte with colors used to indicate the concentration levels of the other osmolytes. The dashed line presented the post-thaw recovery for the single component solution. For sucrose and isoleucine, the post-thaw recoveries of cells cryopreserved in multicomponent solution were consistently higher than those for the single component solution (Fig. 4a,b, e,f). It is also noteworthy that the highest post-thaw recovery of sucrose alone is observed at moderate concentration but the highest post-thaw recovery for SGI was shifted to a lower concentration (146 mM). Glycerol exhibited lower post-thaw recovery for some compositions of SGI than that of the single component (Fig. 4c,d). Isoleucine presented a disorder effect of post-thaw recovery to both sucrose and glycerol (Fig. 4b,d). Unlike single component studies, the variation in post-thaw recovery with composition rose and fell over the parameter space.

#### Raman spectroscopy of Jurkat cells cryopreserved in single and multicomponent solutions

**Figure 5:**
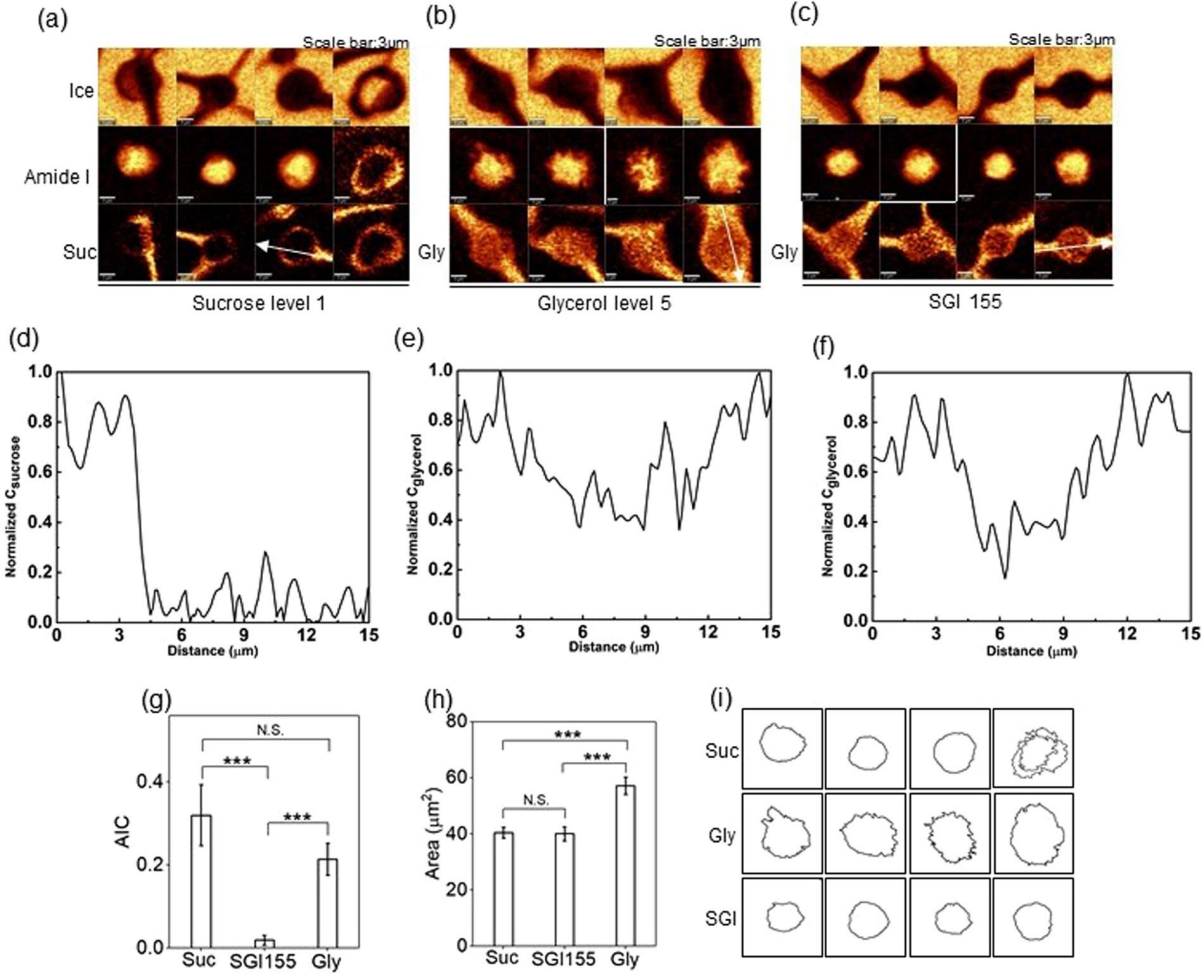
(**a**) Raman images of ice, amide I, and sucrose of cells cryopreserved in 146 mM sucrose solution. (**b**) Raman images of ice, amide I, and glycerol of cells cryopreserved in 10% glycerol solution. (**c**) Raman images of ice, amide I, and glycerol of cells cryopreserved in SGI155 solution. (**d**) Normalized concentration of sucrose along the white arrow in (**a**). (**e**) Normalized concentration of glycerol along the white arrow in (**b**). (**f**) Normalized concentration of glycerol along the white arrow in (**c**). (**g**) AIC of cells cryopreserved in 146 mM sucrose solution, 10% glycerol solution and SGI155 solution (n = 8, p = 0.1253 between Sucrose (Suc) and Glycerol (Gly), p = 0.0002 between Suc and SGI155, p = 0.0009 between Gly and SGI155). (**h**) Cross-sectional area of cells cryopreserved in 146 mM sucrose solution, 10% glycerol solution and SGI155 solution (n = 8, p = 0.0004 between Suc and Gly, p = 0.4504 between Suc and SGI155, p = 0.0007 between Gly and SGI155). (**i**) Cell boundary of cells cryopreserved in 146 mM sucrose solution, 10% glycerol solution and SGI155 solution.

Post-thaw recovery of cells frozen in SGI solution was generally higher than that in single component solutions. In order to understand the difference, cells were cryopreserved in 146 mM sucrose solution (sucrose level 1), 10% glycerol solution (glycerol level 5), or combination of 146 mM sucrose, 10% glycerol and 46 mM isoleucine solution (SGI155) at a constant cooling rate of 1 °C/min down to −50 °C, and typical Raman images rendered on the signals associated with ice, amide I, sucrose or glycerol were generated (Fig. 5a–c). Normalized concentrations of sucrose and glycerol determined using spectroscopy showed that sucrose was present in the extracellular space (and not the intracellular) (Fig. 5d). Glycerol however was present both inside and outside the cell for cells cryopreserved in 10% glycerol (Fig. 5e) and SGI155 (Fig. 5f), respectively.

Cells cryopreserved in 146 mM sucrose solution displayed both small ice crystals and/or large pieces of ice. On the contrary, only small ice crystals were formed in cells cryopreserved in 10% glycerol solution. For cells cryopreserved in SGI155 solution, little IIF was observed. The AIC of cells cryopreserved in single component solution was significantly greater than that of cells cryopreserved in multicomponent solution (Fig. 5g). In contrast with sucrose, Raman images of glycerol showed considerable penetration of glycerol into all frozen cells. It was noteworthy that cells cryopreserved in single component glycerol solution appeared in larger size (57 µm^2^) than those cryopreserved in solutions containing sucrose (41 µm^2^) as well as SGI155 (40 µm^2^), suggesting lower water content for cells in the multicomponent osmolyte solutions (Fig. 5h). Cells cryopreserved in single component glycerol solution also showed irregularities on the cell membrane consistent with blebbing (Fig. 5i).

#### Statistical modeling of multicomponent solutions

**Figure 6:**
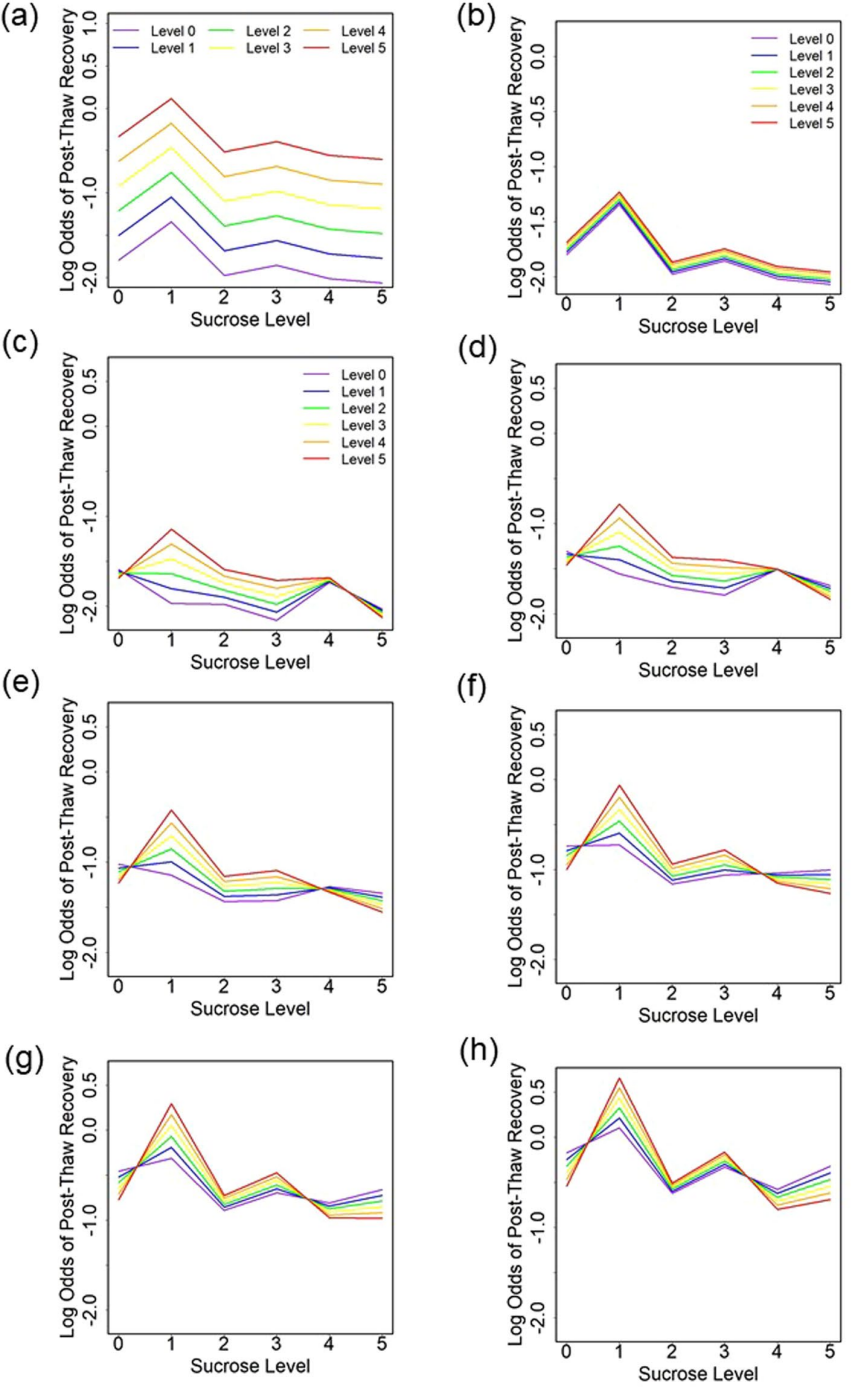
Estimated log odds of post-thaw recovery from the quasi-binomial model without interactions and with coloring by (**a**) level of glycerol and (**b**) level of isoleucine; and estimated log odds of post-thaw recovery from the quasi-binomial model with interactions and coloring by isoleucine level and for a glycerol level of (**c**) 0, (**d**) 1, (**e**) 2, (**f**) 3, (**g**) 4, and (**h**) 5.

The main effects model considered the individual, additive effects of each osmolyte without interactions. It showed that post-thaw recovery was dominated by increasing glycerol level (Fig. 6a), while increasing the isoleucine level only led to small improvement (Fig. 6b). Increasing glycerol by one level was associated with 34% higher odds of post-thaw recovery (95% CI: 29–33% higher; p < 0.001). Increasing isoleucine by one level was associated with 3% higher odds of post-thaw recovery (95% CI: 0–6% higher; p = 0.09). Sucrose had a statistically significant effect on post-thaw recovery (p < 0.001) with its effect peaking at level 1 and then declining (Fig. 6a,b).

We used the interaction model to test for pairwise interactions between osmolytes. There was evidence of interactions between sucrose and isoleucine (p = 0.012) and sucrose and glycerol (p = 0.014). There was no evidence of an interaction between glycerol and isoleucine (p = 0.36). For the interaction model, we visualize the impact of the osmolyte levels on the estimated log odds of post-thaw recovery (Fig. 6c–h). We see that more isoleucine is generally better unless there is a high level of sucrose, in which case isoleucine degrades the post-thaw recovery. The overall post-thaw recovery was proportional to glycerol levels, but the trends were distinct within glycerol levels (Fig. 6c–h). For example, the variation of post-thaw recovery between isoleucine levels was negligible for sucrose level 2 and glycerol level 5 (Fig. 6h) in comparison to variation for the same sucrose level and glycerol level 0 (Fig. 6c). Lastly, the best post-thaw recovery is estimated to be for sucrose level 1 and isoleucine level 5 for all glycerol levels, which is consistent with experimental data. Glycerol was estimated to always have a positive association with post-thaw recovery, though the size of effect varied based on the level of sucrose (see Supplementary Fig. S4).

**Figure 7:**
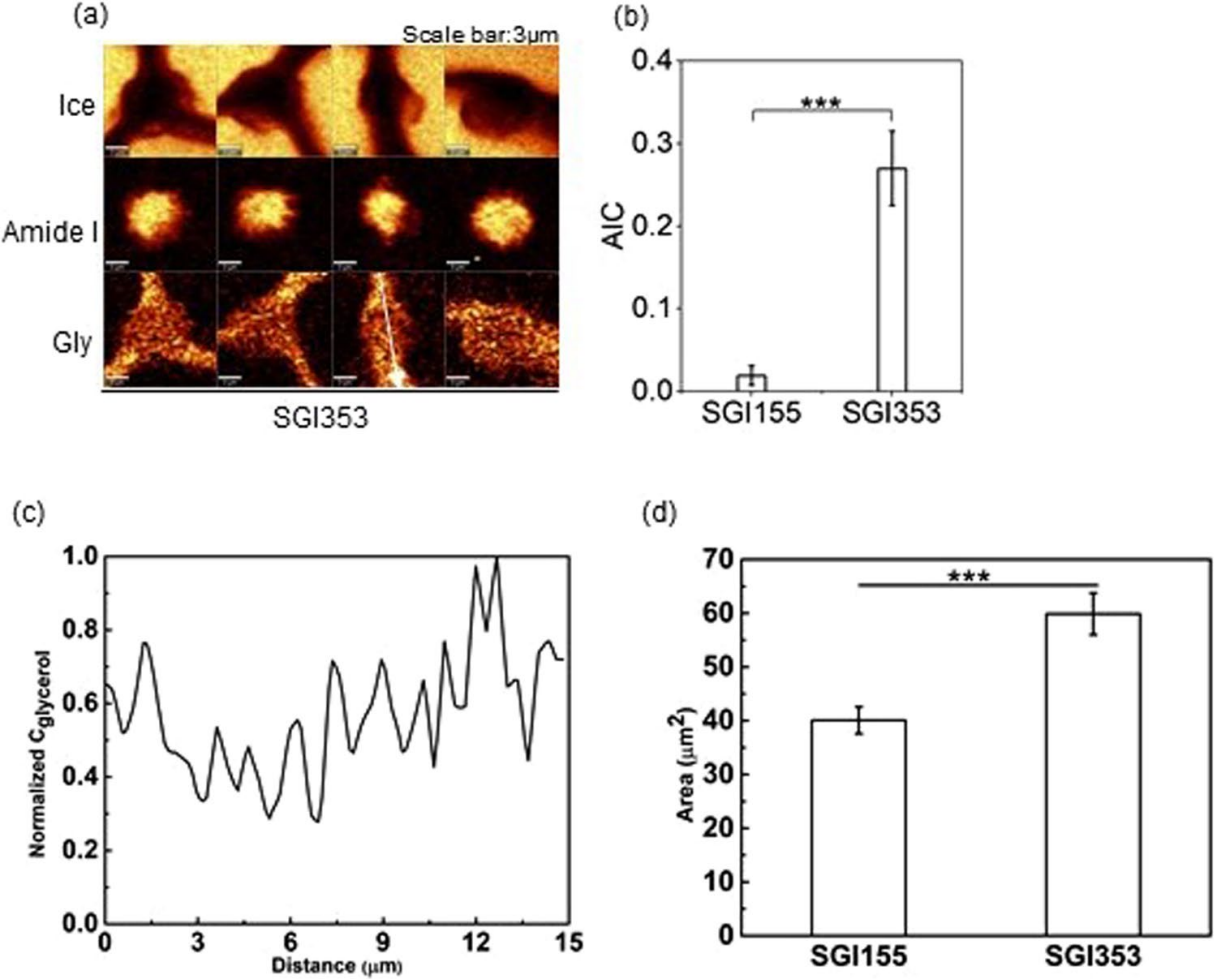
(**a**) Raman images of ice, amide I, and glycerol of the cells cryopreserved in SGI353 solution. (**b**) AIC between cells cryopreserved in SGI155 and SGI353 solution (n = 8, p = 0.0001). (**c**) Normalized concentration of sucrose along the white arrow in (**a**). (**d**) Cross-sectional area of the cells cryopreserved in SGI155 and SGI 353 (n = 10, p < 0.001).

Raman images of ice, amide I and glycerol for the cells cryopreserved in SGI155 (i.e., optimal) and SGI353 solution were generated and were consistent with the conclusions of the statistical model (Fig. 7a). More IIF was observed in the cells cryopreserved in solution SGI353 solution than the cells in SGI155 solution, accordingly, AIC of cells cryopreserved in SGI353 solution was greater than that of the cells in SGI155 solution (Fig. 7b). Normalized glycerol concentration determined using Raman spectroscopy revealed that glycerol was also present inside the cells (Fig. 7c). However, the cross-sectional area of cells cryopreserved in SGI155 (40 µm^2^) was significantly smaller than SGI353 (60 µm^2^) (Fig. 7d).

## Discussion/Conclusion

There has been tremendous interest in the replacement of DMSO. Trehalose, other sugars and specialty polymers have been studied as replacements for DMSO^21–25^. Glycerol has been used to preserve red blood cells^26,27^. None of these studies have found a single molecule capable of replacing DMSO. Osmolyte mixtures have been used for protein stablization^28–30^ but have not been used for cryopreserving cells. This work used osmolyte mixtures to improve the post-thaw recovery of Jurkat cells, which was consistent with our previous study using mesenchymal stem cells^10,17^

It has long been known that water content inside the cell is an important factor in cell response during freezing^31^. In this investigation, cell size is noted as a surrogate for intracellular water content. As noted in the results, the cell size varied between the different single and multicomponent solutions tested. The presence of sucrose in a solution resulted in small cell size and therefore low intracellular water content. It is noteworthy that in Fig. 5, the area of the cells in 146 mM sucrose and SGI155 were roughly the same but the AIC for the cells in the sucrose solution was very high (~0.3) with little or not ice found in the cells frozen SGI 155. Therefore, cell area/water content alone does not correlate with freezing response. Cells in the presence of glycerol alone or higher levels of sucrose exhibited larger cell sizes and therefore higher water content. In the case of the larger cell size for SGI353, the presence of intracellular ice increased the cell volume measured.

**Figure 8:**
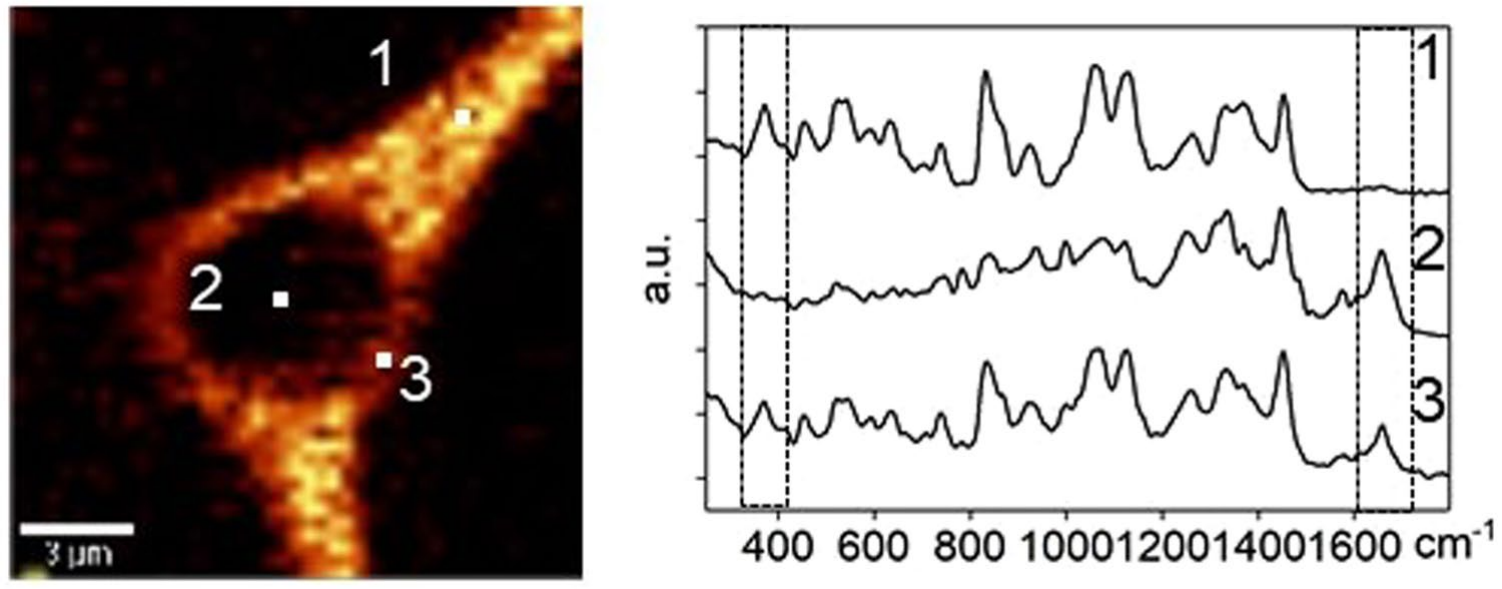
Raman spectra of an extracellular spot (labeled 1), an intracellular spot (labeled 2) and a third spot at interface between cell and extracellular ice (labeled 3) for a cell cryopreserved in 730 mM sucrose solution.

The outcome of this investigation and other studies can be used to understand molecular mechanisms of action for the osmolytes. It has long been hypothesized that disaccharides such as trehalose and sucrose could lower the transition temperature of membranes by replacing the water molecules in lipid headgroups^32–34^, or by vitrification of the stabilizing solutes^35^. The spatial distribution of osmolytes was examined using a cell cryopreserved in 730 mM sucrose solution. The Raman spectra of three spots were selected from the Raman images (Fig. 8). The Raman spectra of spot 1 showed a strong peak of sucrose but no peak for amide I, which suggested spot 1 was extracellular. On the contrary, the Raman spectra of spot 2 showed a strong peak of amide I but no peak of sucrose, which demonstrated that sucrose did not penetrate the cell and this spot was in the cell interior. However, both signals of amide I and sucrose were detected from the Raman spectra of spot 3, indicating that the sucrose and cell had overlap on the barrier between extracellular and intracellular, the cell membrane. The observed phenomenon was consistent with long-held theory that the protective properties of sucrose partially result from its interaction and stabilization of membranes and consistent with other Raman studies of sugars and cell membrane interactions^36^. A recent study has found that non-penetrating cryoprotectants can also provide protection^37^ suggesting that stabilization of the cell membrane may be critical for post thaw recovery.

Sugars such as sucrose also interact with water. Sucrose has been shown to have a destructuring effect on the water tetrahedral hydrogen bond network has been observed in both experimental studies and molecular dynamics simulations^38,39^. For high concentration sucrose solutions, it was found that all the water molecules were involved in hydrogen bonds with sucrose^12^, and that the hydrogen bonds formed between sucrose and water significantly slowed down the water dynamics^40^. The interaction between sucrose and water can manifest on a macroscale. Bailey and colleagues found that the addition of sucrose to dimethyl sulfoxide changed the ice crystal patterns observed upon freezing^41^.

The statistical model suggests that glycerol plays a major role in cell survival and interactions between glycerol and sucrose influence post thaw recovery as well. The influence of glycerol on cell survival has been known for over 60 years^42^. Glycerol has long been associated with stabilization of proteins^43^. As demonstrated in Figs 5 and 7, glycerol penetrates the cell membrane and provides a stabilizing benefit in the intracellular space. The importance of penetrating cryoprotectants on post-thaw recovery has long been known^31^.

As with sugars, the results in this study suggest that sugar alcohols act on water molecules. Previous studies have shown that the hydrogen bonding between glycerol and water plays a significant role to inhibit ice crystallization and the structure of ice crystals formed during freezing^13,44–46^. A recent study demonstrated changes in the structure of ice formed in the presence of different sugar alcohols^47^. The result of this investigation is consistent with those previous studies.

Interactions between sucrose and isoleucine determined with the statistical model are consistent with the observation by Wen and colleagues that the presence of specific proteins actually stabilizes trehalose during freezing and prevents precipitation^16^ and suggest an important role in the solution.

## Electronic supplementary material

Dataset 1
