# Supplementary material for: Characterizing the “sweet spot” for the preservation of a T-cell line using osmolytes
Source: Sci Rep. 2018 Nov 1;8:16223. doi: 10.1038/s41598-018-34638-7 (PMC6212455; doi:10.1038/s41598-018-34638-7)
Supplement: Supplementary file 1 — Dataset 1 [file 41598_2018_34638_MOESM1_ESM.pdf]

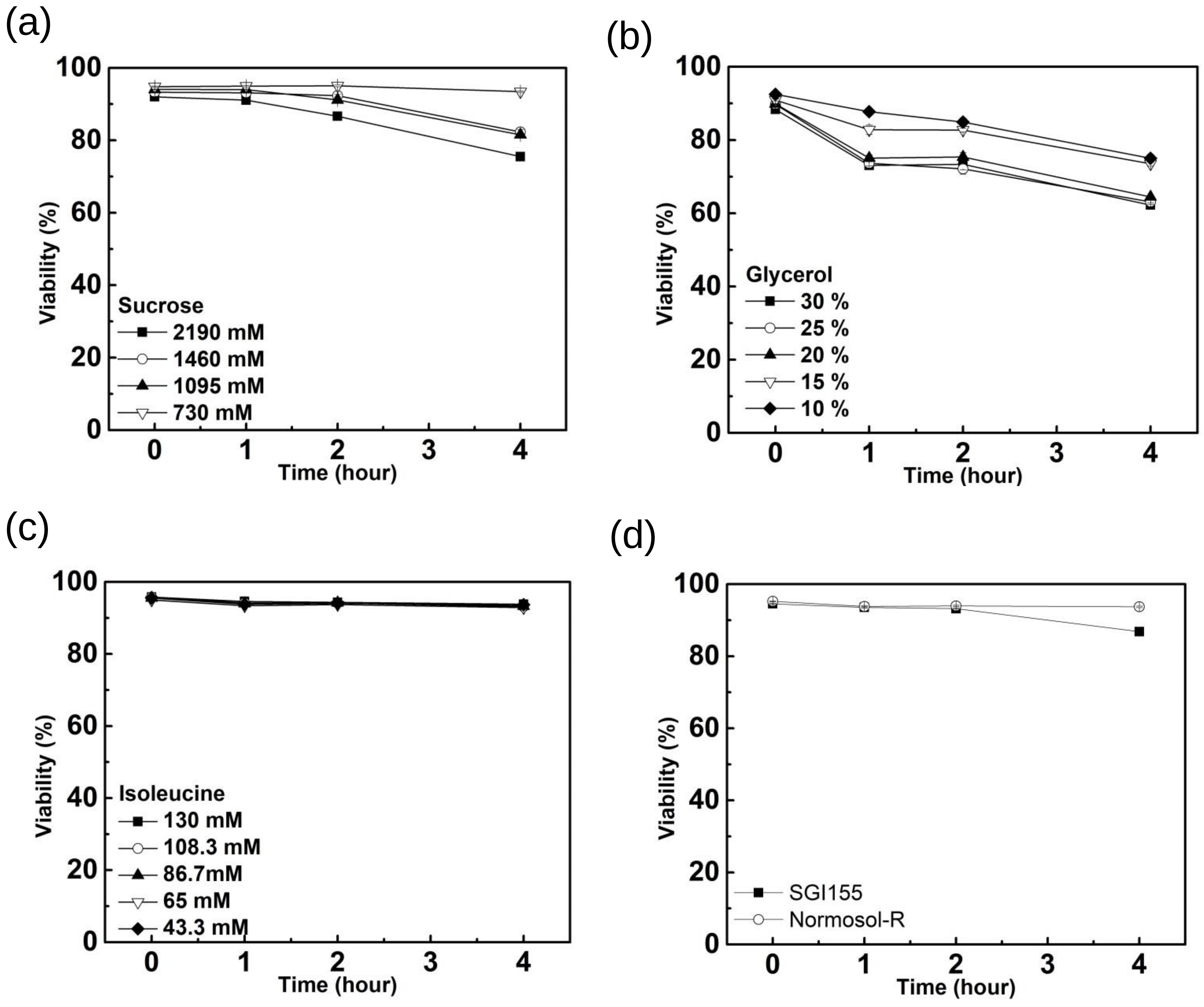

Supplementary Figure. S1 Viabilities of Jurkat cells incubated at different time points post exposure in different concentrations of (a) sucrose, (b) glycerol, (c) isoleucine and (d) Normosol-R and SGI155.

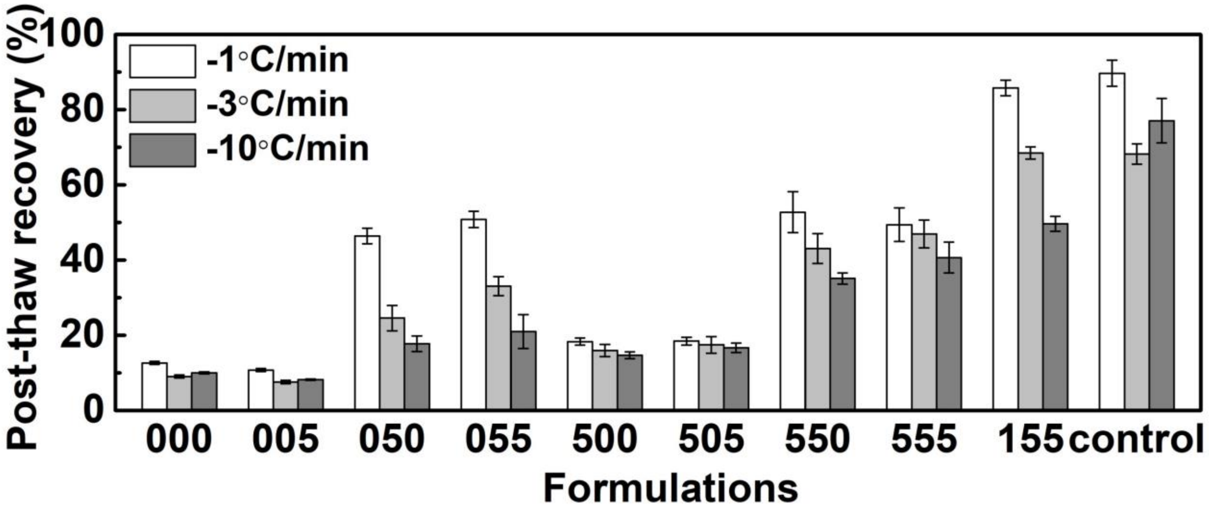

Supplementary Figure. S2 Post-thaw recoveries of 8 formulations in the corners of the parameter space (level 0 or level 5 of a given component), the optimal formulation (SGI155) and 10 % DMSO control as a function of three cooling rates (-1 °C/min, -3 °C /min and -10 °C /min)

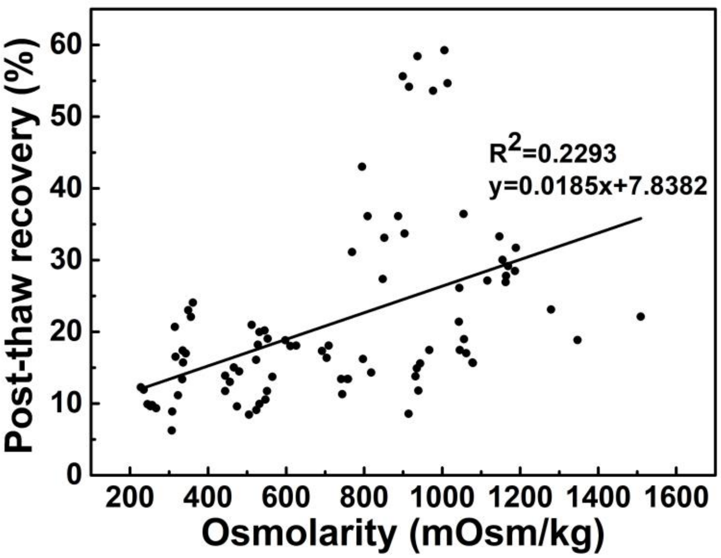

Supplementary Figure. S3 Post thaw recovery of Jurkats cryopreserved at 1 °C/min as a function of cryoprotectant osmolarities

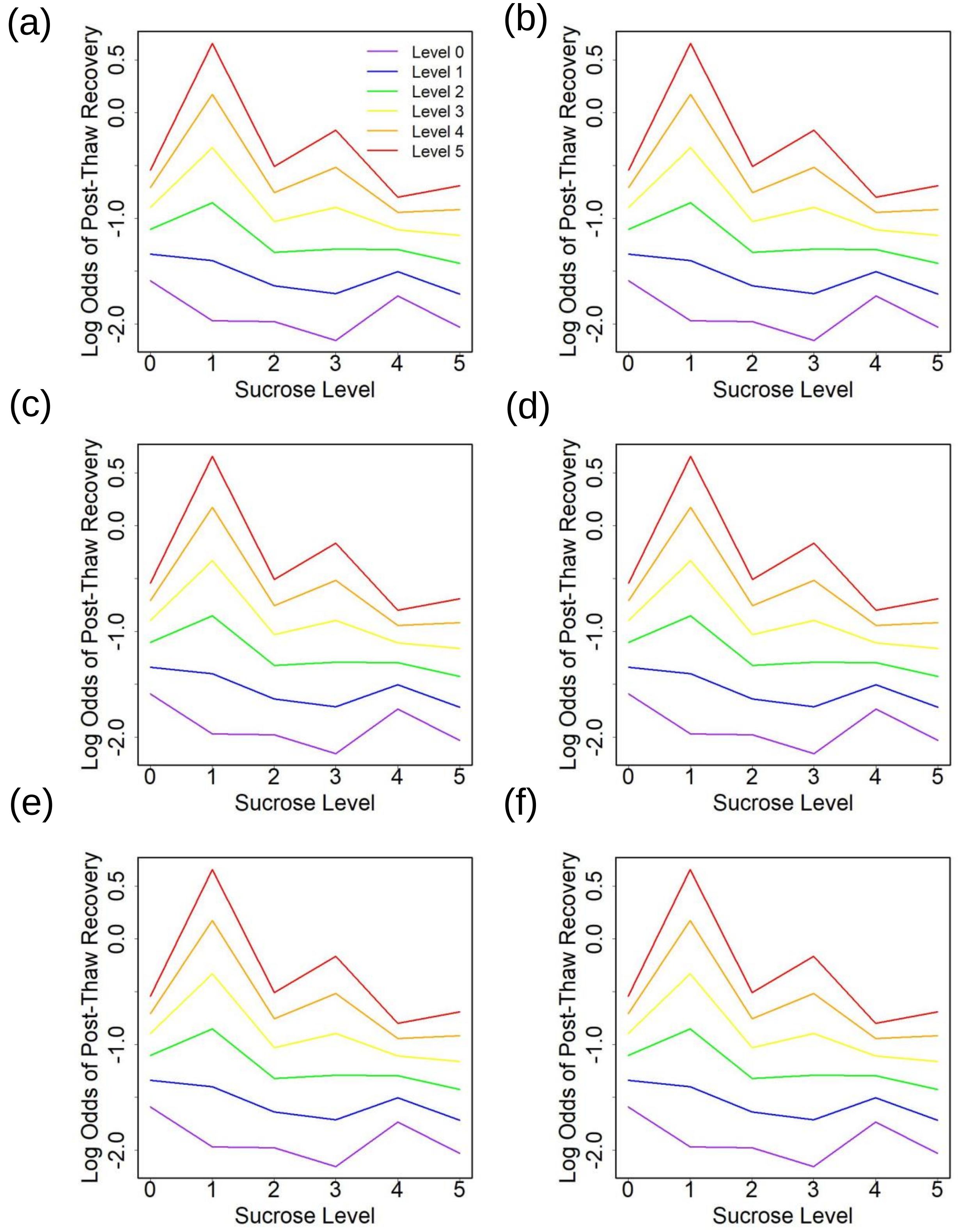

Supplementary Figure. S4 The estimated log odds of post-thaw recovery from the quasi-binomial model with interactions and coloring by glycerol level and for an isoleucine level of (a) 0, (b) 1, (c) 2, (d) 3, (e) 4, and (f) 5
